# Statin use is associated with a reduced incidence of colorectal cancer: a colonoscopy-controlled case–control study

**DOI:** 10.1186/1471-230X-12-36

**Published:** 2012-04-24

**Authors:** Thomas Broughton, Jamie Sington, Ian LP Beales

**Affiliations:** 1 Gastroenterology Department, Norfolk and Norwich University Hospital, Norwich, NR4 7UZ, UK; 2 Histopathology Department, Norfolk and Norwich University Hospital, Norwich, NR4 7UZ, UK; 3 Norwich Medical School, University of East Anglia, Norwich, NR4 7TJ, UK

**Keywords:** Aspirin, Chemoprevention, Hydroxymethylglutaryl-CoA reductase inhibitors, Colorectal adenocarcinoma

## Abstract

**Background:**

The aetiology of colorectal cancer (CRC) remains elusive in the majority of cases. There is experimental evidence to show that HMG-CoA reductase inhibitors (statins) may inhibit proliferation and induce cause apoptosis in CRC cells and although some clinical studies have suggested that statins may protect against the development of CRC, this has not been a consistent finding. Therefore we have examined any potential protective effects of statins by comparing statin use in patients with colorectal cancer against a control group.

**Methods:**

This was a case–control study examining statin use in symptomatic patients attending for diagnostic colonoscopy. Statin use was compared between patients with CRC and a control group, who had all had normal colonoscopy. Structured interviews and clinical records notes were used to determine drug exposure. Logistic regression was used to compare statin exposure and correct for confounding factors.

**Results:**

There was a significant inverse association between previous statin use and a diagnosis of CRC (OR = 0.43 (95% confidence interval 0.25 – 0.80), p<0.01). This inverse association was stronger with higher statin doses (OR = 0.19 (0.07 – 0.47), p<0.01) and greater duration of statin use (statin use >years: OR = 0.18 (0.06 – 0.55), p<0.01).

**Conclusions:**

Statins use was associated with a protective effect against the development of CRC. This effect is associated with a significant dose and duration response. These findings need to be repeated in other observational studies before an interventional study can be considered.

## Background

Colorectal cancer (CRC) is common, with an incidence of 20.1/100,000/year in men and 14.6/100,000/year in women [1]. Worldwide, the incidence of CRC ranks fourth in frequency in men and third in women. CRC accounts for over 1 million new cases of cancer each year, 9.4% of the world’s total [2]. The prevalence is second only to that of breast cancer worldwide, with an estimated 2.8 million people alive with bowel cancer within 5 years of diagnosis [1,2]. The disease causes over 592,000 deaths each year [1]. Treatment involves surgical resection of the bowel in over 80% of patients. Adjuvant chemotherapy may reduce local recurrence and mortality but is dependent on the stage of the cancer. Fewer than 50% of patients survive more than 5 years after diagnosis [1,3]. Although improved surgical and oncological techniques and population-wide screening programmes have certainly had a positive impact on the incidence and outcomes from CRC, further benefits in outcomes should be possible with chemopreventative strategies. At present there are no widely accepted chemo-preventative interventions. Inhibition of cyclooxygenase-2 (COX-2) has been associated with a reduced incidence of CRC in case control studies and experimental animal studies have given encouraging results [4]. However although COX-2 inhibitors do reduce adenomatous polyp formation, the adverse cardiovascular profile of these drugs, will undoubtably prevent their use in a wide chemopreventative strategy [5]. The potential for cardiovascular safety needs to be considered in a prevention strategy, especially as many of the risk factors such as obesity and hyperinsulinaemia are common to both vascular disease and CRC [6]. Although long-term use of aspirin does seem to reduce the incidence of CRC, it is not currently recommended because of the increased risk of bleeding [7].

Against this background, the effects of HMG-CoA reductase inhibitors (statins) are particularly pertinent. On one hand they could be expected to be commonly prescribed to patients at risk of colon cancer, hence any effects on increasing CRC risk may be clinically important and on the other, there are data suggesting a potentially important protective effect of statins against CRC [8-12]. Laboratory studies have shown that statins induce apoptosis, inhibit proliferation and reduce invasion in multiple colon cancer cell lines [13,14]. This effect seems to be a class effect and has been documented with lovastatin, simvastatin, pravastatin and atorvastatin and is most plausibly due to the inhibition of the mevalonate synthetic pathway reducing the cellular availability of substrates required for the isoprenylation of small signaling G-proteins and the resultant inhibition of pro-carcinogenic and pro-survival pathways [13-15]. Other protective mechanisms have been suggested, including anti-inflammatory actions, reduction in circulating lipids and a beneficial effect on adipokine secretion profiles [16].

Clinical studies with statins and CRC have given variable results. Two separate studies showed a highly significant 50% reduction in CRC incidence in statin users [8,11]. A more recent observational study showed a 38% reduction in CRC incidence only in lovastatin users with a non-significant trend for greater protection with more than 3 years therapy [17] and two separate meta-analyses suggested a protective effect of statins [9,18] Bardou *at al* confirmed that randomized controlled trials tended to show a small a non-significant-reduction in CRC incidence in statin users, whilst observational and case–control studies suggested a persistent but modest protective effect of statins [18]. These differences are thought to arise from the low absolute incidence of CRC in the randomized trials and relatively limited short term nature of statin trials, which were not primarily designed to examine cancer incidence. In addition to lovastatin protective effects have been reported with simvastatin and pravastatin [8,19]. However other studies, often with different methodologies have shown no effects [19-25]. The reasons for these discrepancies are not clear but probably involve differences in the study design, populations, specific statin use in different populations, age at initiation of statin use and the controls examined, duration of statin exposure and exposure to confounding factors. However, many of the recent observational studies had significant limitations with statin use being measured from prescriptions and an uninvestigated control group recruited from general practice databases. In several of the studies statin exposure was regarded as positive if as little as one prescription or 3 months therapy was taken [24,25]. The maximal duration of follow up in cohort studies was usually less than 5 years and this may not have been sufficient time for the effects of statins to become apparent as other studies with over 5 years, or a mean of 9 years follow up showed more protective effects [8,10]. A recent longer-term study showed that > years statin exposure, assessed using a questionnaire, was not associated with a reduced incidence of colon cancer [26]. Statins and other medications purchased over the counter were not included in many of the studies and neither BMI nor smoking data were available in all studies. The controls were not investigated with endoscopy so there was no certainty over these diagnoses and no information available to assess early CRC. One major review confirmed that the data on statins and colon cancer were conflicting and inconsistent and although a number of studies have provided no evidence of protection, the situation was sufficiently unclear that further clinical studies were warranted [19].

Therefore, given this uncertainty we have examined the effect of statin use on the incidence of CRC in an average risk United Kingdom population.

## Methods

### Study design

This study was conducted as a retrospective case–control study. Information was obtained from brief structured patient interviews and was verified through subsequent review of the clinical notes and past referral letters. Where there was incongruence between prescriptions indicated in the clinical notes and that relayed by the patients, the patients’ answer was taken as the most accurate description of drugs being taken. A history of the patient’s statin use was gathered, including the dose, duration and type of statin that was used. Statin use in the 6 months prior to diagnosis was excluded because the potential chemopreventative effects of statins may not have materialized after such short term use. Information regarding exposure to other known risk factors was also collected using the standardized interview. Regular use of aspirin or NSAIDs was defined as one dose per week or more. The interviews lasted 15 minutes and were conducted in a private interview room.

### Study population

All the patients were under the care of the Gastroenterology Department at the Norfolk and Norwich University Hospital. This is a large University Teaching Hospital serving a predominantly rural population of approximately 600 000 people. The stability of, and consistent referral patterns within the catchment population has been noted in previous epidemiological studies [27] and drug histories could almost always be verified by clinical notes.

### Controls

Patients attending for their first diagnostic colonoscopy from 1/09/2009 to 31/5/2010 were invited to participate. Patients having a colonoscopy for surveillance for inflammatory bowel disease or previous adenomatous polyps or cancer were excluded as were those where the indication was a screening colonoscopy in asymptomatic patients for a high risk family history, acromegaly or previous uretosigmoidostomy. All patients were aged over 18, fluent in spoken English and underwent a structured interview prior to having a diagnostic colonoscopy. For this study, controls were those found not to have either CRC or adenomatous polyps. Subjects were excluded from further analysis if excised polyps were not recovered for histological analysis, or there was no histological confirmation of polypoid lesions when expert colonoscopists left presumed hyperplastic polyps *in situ*.

### Cases

Patients who were subsequently found to have CRC at their post-interview colonoscopy were included, in addition patients with CRC diagnosed in the same time frame but who did not have a pre-colonoscopy interview, were indentified and underwent the same structured interview after diagnosis. Post-diagnosis interviewing was utilized for purely practical reasons: given the number of procedures performed in our unit and the limited time available to him, the part-time student interviewer had insufficient time to interview all patients pre-colonoscopy. As the majority of diagnostic colonoscopies have non-malignant findings, it was necessary to use additional methods to ensure an adequate sample size. Cases interviewed post-diagnosis were on exactly the same referral, diagnostic and treatment pathway as all other cases and controls. All cases had histological confirmation and were reviewed by a specialist GI pathologist (JS).

### Ethics and research governance

All patients gave written informed consent and the study was approved The Norfolk Research Ethics Committee and the Norfolk and Norwich University Hospital Trust Research Governance Committee.

### Sample size and statistical analysis

An initial sample size of at least of 93 cases and 93 controls was planned: this had 80% power to detect a 50% relative risk reduction in CRC incidence in statin users, assuming a statin use rate of 40% in controls.

Statistical analyses were performed using SPSS version 16.0. The percentage of participants that had previously used statins in each case group was compared against the control group using a chi square statistic. The differences between the groups were quantified using the calculated odds ratios and 95% confidence intervals, with the significance level set at p<0.05. Further analysis was performed, comparing the differences in duration, dose and type of statin use between each case group and the control group. The duration of statin use was characterised into 3 categories: use was characterised into 3 categories <2 years, 2–5 years and >5 years. The statin dose category was dichotomised into low dose (dose category was dichotomised into low dose 40 mg simvastatin or equivalent /day) and high dose (40 mg simvastatin or more or equivalent / day). The type of statin used was separated into 2 categories, simvastatin and other types of statin. For every statin variable, each case group was compared against the control group and odds ratios were calculated with 95% confidence intervals compared to the reference never used statin. All odds ratios were recalculated using unconditional logistic regression, correcting for potential confounding factors.

## Results

### Baseline characteristics and confounding factors

A total of 101 patients with CRC and 132 controls were included in the study. Baseline characteristics and demographic information for cases and controls are shown in Table 1. There were significant differences (p = 0.05) between the groups with regard to age, gender, current weekly alcohol intake and type II diabetes. Table 2 shows that previous aspirin and metformin use were both significantly more common in controls than cases. There were no significant differences between the cases and controls with regard to the use of NSAIDs, calcium channel blockers and other diabetes medications. The multivariable regression analyses adjusted for all potential confounding factors that showed statistically significant differences between the groups. This included age, gender, type II diabetes, weekly alcohol intake, and a history of aspirin or metformin use. The indications for the diagnostic colonoscopy are shown in Table 2. The indications were typical of those in a United Kingdom general hospital and were broadly similar between cases and controls. Rectal bleeding was significantly more commonly the indication in cancer patients and a change in bowel habit was significantly more common in those with a normal colonoscopy. Anaemia was found to be a slightly, but not significantly, more common indication in the control group.

**Table 1:** Baseline characteristic

	**Normal**	**CRC**
Number	132	101
Male (%)	61 (46.2)	63 (62.4)
Mean age, y (SD)	63.8 (12.2)	70.3 (10.2)
Type II diabetes (%)	26 (19.7)	8 (7.9)
Inflammatory bowel disease (%)	6 (4.5)	2 (2.0)
Family history* (%)	19 (14.4)	9 (8.9)
Smokers† (%)	55 (41.7)	44 (43.6)
Established coronary artery disease**	10 (8)	5 (5)
Mean smoking, pack years (SD)	8.3 (12.6)	8.4 (12.0)
Alcohol drinkers† (%)	89 (67.4)	76 (75.2)
Mean alcohol, units/week‡ (SD)	6.5 (7.6)	8.5 (7.3)
BMI (SD)	27.1 (4.4)	26.2 (4.0)
HRT (% of women)	9 (12.7)	8 (21.1)
Mean parity (% of women)	2.25 (1.13)	2.58 (1.11)
Number of parous women (%)	65/71 (91.5)	37/38 (97.4)

Baseline characteristics of subjects included in the study.

Abbreviations: BMI = Body Mass Index; CRC = colorectal cancer, HRT = Hormone Replacement Therapy.

* 1^st^ degree relative with family history of CRC.

† Current or ex-smokers / alcohol drinkers.

** Coronary artery disease confirmed by angiography, cardiological opinion or documented previous acute coronary syndrome or by-pass grafting.

‡ Current weekly alcohol consumption.

**Table 2:** Indications for colonoscopy

	**Controls**	**CRC cases**	***p *value**
	**n (%)**	**n (%)**	
Anaemia			
Total	41 (31)	20 (20)	0.06
Statin users	22	4	0.01
Positive FOB screening			
Total	26 (19)	13 (13)	0.17
Statin users	14	3	0.08
Rectal bleeding			
Total	7 (5)	17 (16)	0.01
Statin users	3	5	0.60
Abnormal or equivocal imaging			
Total	4 (3)	16 (16)	0.01
Statin users	1	2	0.60
Change in bowel habit			
Total	41 (31)	25 (25)	0.29
Statin users	15	5	0.86
Abdominal pain			
Total	13 (10)	8 (8)	0.62
Statin users	3	1	0.62
Palpable abdominal mass			
Total	0 (0)	2 (2)	0.33
Statin users	0	0	-

### Statins

There was a highly significant inverse association between previous statin use of at least 6 months duration and a diagnosis of CRC. Table 3 shows an extremely strong inverse association after adjustment for potential confounders [OR = 0.43 (0.25 – 0.80), p<0.01]. There were no patients in our cohort that had taken statins previously but had subsequently stopped sometime before having the colonoscopy. Thus statin exposure reflects a continuous period prior to diagnosis. The majority of statin use was prescribed in primary care for primary prevention of coronary artery disease. There was no difference in reported statin use between those cancer patients interviewed pre-diagnostic colonoscopy (13/64, 20%) and those interviewed having already been informed of their diagnosis (7/37, 18.9%) There was no obvious difference in statin use between cancers of the rectum (17%), left colon (20%) or right colon (22%), but numbers in each group were too small to analyze separately. Similarly there was no significant difference in statin use when the stage of colorectal cancer was examined although there was a trend for statin use to be less common in more advanced disease (Dukes’ A 20% , B 21.5%, C 18% and D 12.5%).

**Table 3:** Use of other medications

	**Control**	**CRC**			
	**n (%)**	**n (%)**	**OR**	**(95% CI)**	**p-value**
aspirin	43 (32.6)	15 (14.9)	0.36	(0.18 -.70)	< 0.01
NSAIDs	16 (12.1)	7 (6.9)	0.54	(0.20 -1.35)	0.19
CCMs*	12 (9.1)	7 (6.9)	0.75	(0.27 – 1.97)	0.55
metformin	18 (13.6)	5 (5.0)	0.33	(0.10 -0.89)	0.03
other diabetes	8 (6.1)	1 (1.0)	0.16	(0.03 – 1.19)	0.06

Use of medications in patients with colorectal cancer (CRC) and controls. Results expressed as odds ratio with 95% confidence intervals (95% CI). *calcium channel modulators.

### Duration

The protective effect of statins was greater with increased time of exposure, as shown in Table 4, [<2 years: OR = 0.66 (0.21 – 1.69), p = 0.47], [2–5 years: OR = 0.38 (0.14 – 1.01), p = 0.05], [>5 years: OR = 0.18 (0.06 – 0.55), p<0.01]. There was a significant linear trend for the duration-response relationship between statin use and CRC diagnosis [test for trend, x^2^ (1) = 26.8, p<0.01].

**Table 4:** Statins and CRC

				**Odds ratios (95% CI)**	
	**Controls (%)**	**CRC (%)**	**P-value**	**Unadjusted**	Adjusted*
any previous use	68/132 (51.5)	20/101 (19.8)	< 0.01	0.23 (0.13 – 0.42)	0.43 (0.25 – 0.80)
Duration					
< 2 years	14 (11%)	8 (8)	0.47	0.73 (0.56 – 3.43)	0.66 (0.21 – 1.69)
2 – 5 years	23 (17)	7 (7)	0.05	0.35 (0.15 – 0.86)	0.38 (0.14 – 1.01)
> 5 years	31 (24)	5 (5)	< 0.01	0.17 (0.063 – 0.454)	0.18 (0.06 – 0.55)
Dose					
< 40 mg/day	28 (21)	12 (12)	0.14	0.50 (0.24 – 1.04)	0.51 (0.21 – 1.24)
= or>40 mg/day	40 (30)	8 (8)	< 0.01	0.20 (0.09 – 0.45)	0.19 (0.07 – 0.47)
statin type					
simvastatin	49 (37)	14 (14)	< 0.01	0.27 (0.14 – 0.53)	0.43 (0.10 – 0.73)
other	19 (14)	6 (6)	0.09	0.38 (0.14 – 0.98)	0.49 (0.13 – 1.13)

Use of statins in patients with colorectal cancer (CRC) and controls. Results expressed as odds ratio with 95% confidence intervals (95% CI). *Adjusted for age, gender, type II diabetes, aspirin use, metformin use and weekly alcohol intake and compared to never used statins.

### Dose

There was a dose-dependent inverse relationship between the dose of statin and CRC. The apparent protective effect was greater at the higher statin dosage [OR = 0.19 (0.07 – 0.47), p<0.01] compared to the lower statin dosage [OR = 0.51 (0.21 – 1.24) p = 0.14] which did not show a statistically significant difference. There was a significant linear trend for the dose–response relationship between statin use and CRC diagnosis [test for trend, x^2^ (1) = 25.5, p<0.01](Table 4).

### Type of statin

Simvastatin was the most commonly used statin (75/106; 70.8%) the distribution of statin use was comparable between cases and controls (Table 5). Statin use was separately anaylsed as simvastatin and other statins. Cancer patients were significantly less likely to have used simvastatin than controls [OR = 0.43 (0.10 – 0.73), p<0.01] but although there was a similar numerical effect for other statins, given the smaller numbers, this did not reach statistical significance [OR = 0.49 (0.13 – 1.13), p = 0.09].

**Table 5:** Use of statins

	**Control (%)**	**CRC (%)**
simvastatin	49 (37.1)	14 (13.0)
atorvastatin	7 (5.3)	3 (3.0)
pravastatin	4 (3.0)	1 (1.0)
rosuvastatin	8 (6.0)	2 (20.0)
Total	68 (51.5)	20 (19.8)

Use of different statins in patients with colorectal cancer (CRC) and controls.

### Statins and aspirin

Aspirin use was significantly protective against CRC [uncorrected OR = 0.36 (0.19 – 0.70) p<0.01]. However, when adjusted for statin use these protective effects became non-significant against CRC [corrected OR = 0.91 (0.39 – 2.11) p = 0.83]. However, after adjustment was made for aspirin use, the protective effects of statins remained significant against CRC [corrected OR = 0.42 (0.12 – 0.71) p<0.01]. The combination of aspirin and statin was associated with a numerically, but statistically insignificantly, greater protective effect against CRC than either statin or aspirin alone [corrected OR 0.15 (0.04 – 0.47)].

## Discussion

In this case–control study, statin use was associated with a significantly reduced incidence of CRC. These findings remained after adjustment for potential confounding factors. There was a significant duration- and dose–response relationship with greater statin exposure offering more protection against CRC. The use of simvastatin was significantly protective but although exactly the same pattern was seen with the other statins, the numbers were too small to reach conventional statistical significance. These data support a causal relationship between statin exposure and reduced risk of colorectal neoplasia. These results are consistent with previous studies and a meta-analysis suggesting a protective effect [8-12], and the protective effective against CRC seen in the original randomized trials of statin use in cardiovascular disease [18,28], although the strength of the apparently protective effect is rather greater in our study than several other positive studies. Not all case–control and cohort studies have shown consistent results and no association with reduced risk has been reported [20,22-25]. There may be several reasons for the inconsistencies between studies, including different populations with different underlying CRC risk, different control groups, and inadequate length of follow up in cohort studies or insufficient statin exposure to detect a protective effect (some studies including all patients with statin exposure of only 3 months).

There is biological plausibility to our data: we detected both time- and dose-dependent effects against CRC development and experimental studies are supportive of CRC-protective effects of statins. In addition to *in vitro* cell line studies, statins have been shown to reduce polyp formation in Min^−/−^ mouse models [29] and pre-neoplastic and neoplastic lesions in animal models [30,31]. A single study has shown that statin use was associated with a reduced rate of post-polypectomy adenomatous polyp formation [32], although another study with shorter follow up failed to detect any protective effect of statins against polyp recurrence [33].

More recent studies have suggested that biological differences may underlie some of the variability in the apparent effects of statins. Upregulation of HMG-CoA has been reported in CRCs but one study has shown this to be more marked in left sided cancers [15]. Similarly HMG-CoA genotype seems to influence the protective effect of statins: a higher activity allele was associated with a protective effect, whereas a significant protective effects was not associated with a lower activity allele [11]. The polyp-cancer sequence is estimated to take 10–15 years and it is not clear, where along this sequence the effect of statins may be most noticeable [33]. Experimental studies have shown that statins may become less effective in cell lines as more genetic and hence functional changes downstream of the putative cellular effect of statins accumulate [34,35]. If, for example, the effect of stains is more prominent at stages of polyp-development or progression, then the average age of initiation of statin therapy in any population is likely to contribute to differing effects of statins on CRC incidence between studies.

The main specific strengths of our study are the comprehensive drug history available and the fact that all patients underwent diagnostic colonoscopy. Many previous studies have used prescribing records and although these studies have many advantages, they may misreport actual drug exposure as not all prescribed medication is actually consumed. In addition in the United Kingdom aspirin, ibuprofen, diclofenac and simvastatin are available to purchase without prescription, thus we feel our records of drug exposure are both accurate and comprehensive. Asymptomatic colon cancer is not uncommon and our use of a control group, who all underwent colonoscopy will have minimized bias due to misdiagnosis which is a problem in uninvestigated cohorts. There are data from the USA showing that patients with co-morbidity undergo more frequent screening colonoscopies [36]. We only included cases and controls having their first ever diagnostic colonoscopy, so removing any potential bias from repeated colonoscopy or previous polyp removal. A potential disadvantage of our design studying symptomatic patients attending for diagnostic colonoscopy is that the control group may exhibit more health-seeking behavior and be more health conscious and hence introduce bias. However all had genuine symptoms for which colonoscopy would be indicated and as all were provided under the United Kingdom National Health Service, no direct financial or insurance incentives will have influenced decisions regarding colonoscopy. An ideal study would examine statin use in cases and controls from asymptomatic patients invited for colonoscopic colon cancer screening, rather than symptomatic patients; however this would not be possible as the United Kingdom national screening programme is based on initial faecal occult blood screening, followed by colonoscopy only in those testing positive. Interestingly the association of reduced CRC incidence in statin uses was also seen in the sub-group of patients that presented via the national screening program with positive faecal occult bloods, although the overall number of cases in this group was too small to draw definite conclusions (statin use: controls 14/26, 53%, cases 3/13, 23% OR 0.37 (0.06 – 1.17)).

The issue of bias inherent to the control group affecting the results must always be considered in case–control and cohort studies, in particular the possibility that statin users are generally more health-conscious and this explains their lower CRC risk. As with previous studies in this field, we cannot exclude this completely but we feel this is an unlikely explanation of the findings. It is possible that statin-users may have had more specific dietetic input and this lead to a positive change in diet which influenced cancer risk. The majority of statin prescribing was by the patients’ primary care physician for the primary prevention of vascular disease and as such was not governed by any specific protocol and both patient and clinician perception are likely to have influence the use of statins. The control group is comparable to a standard low-moderate CRC risk cohort undergoing diagnostic colonoscopy for any indication in the UK and comparable to the age-matched population as a whole. In terms of smoking, alcohol intake, BMI and type 2 diabetes as markers of health-related behaviors, the control group did not appear to be especially healthier than the cases and the effect of statins persisted after correction for all known confounding variables. The risk of colonic neoplasia is probably increased in both diabetes mellitus and established coronary artery disease [6,37], and this would have been expected to reduce any effects of statins observed in our study. Chronic statin use is associated with a very low incidence of gastrointestinal side effects and so it seems unlikely that statins are over-represented in the control group because they were responsible for symptoms initiating the referral for colonoscopy [38]. It could be argued that because of the proclivity for aspirin to cause gastrointestinal bleeding and anaemia, this could have led to over-representation of aspirin in the control group and indeed there was a slightly higher number of controls in which anaemia was the presenting condition. Whilst this is possible, our study showed a protective effect of aspirin entirely consistent with other studies that used different control groups [25], and the effect of statins persisted after correction for aspirin use. We also showed an apparent protective effect of metformin against CRC, again consistent with previous studies, suggesting our results have external validity [39].

This study did have limitations, some of which were inherent with the case control study design. Despite this study being sufficiently powered to produce statistically significant results, this was a relatively small cohort compared to some similar case control studies. Data collection was cross referenced using patient interviews and clinical notes but recall bias may have caused inaccuracy and difficulty in determining the extent of exposure to medications, although as most subjects were interviewed in the same situation pre-colonoscopy, this is unlikely to unduly influence cases or controls. Data were collected for many potential confounding factors that had been identified from the literature but other uncontrolled confounders could have affected the results. We did not attempt to address the issues regarding diet and cancer risk and further studies in the area would benefit from including this. Although we utilized both pre- and post-cancer diagnosis interviews to establish statin exposure, we do not feel this introduced any bias of recall. All drugs exposures were accurately cross-referenced to medical records (including pre-colonoscopy medical notes), statins are characteristically long-term therapies and once started are very rarely stopped and although patients were informed we were interested in risk factors for their diseases, they were unaware of which particular factors. Our results show no difference in statin use between those interviewed pre- and post-colonoscopy. However the issue of residual confounding by other unrecognized factors must still be considered. Similarly, although adequately powered for our pre-designed end-point, the relatively small size of our study may have contributed to the relatively large protective association that we have described.

Investigating the effect of statins and aspirin produced interesting findings. The use of statins or aspirin produced a significant protective effect, although statin use was considerably more prevalent in this population. There was a suggestion that the combination of aspirin and statin was associated with a lower incidence of CRC, and *in vitro* cell line and mouse models studies would support a beneficial interaction between statins and cyclo-oxygenase inhibitors [14,40-43].

## Conclusion

In conclusion, our case–control study shows that statin use was associated with a lower incidence of colorectal cancer and this effect was associated with a significant dose and duration response. Statins may have a protective effect against the development of CRC. We recommend that future studies examining chemoprevention and causation correct for statin exposure and that as statins do seem to have some promise as chemopreventative agents, further studies examining the effects of statins are required, these should examine the interactions with other agents as well as population or cancer variables that may influence statin response.

## Competing interests

There are no competing interests.

## Authors’ contributions

IB, TB and JS jointly conceived the study and participated in the design of the study, IB and TB carried out the subject interviews and collected the data. JS performed the histopathological review of the specimens. TB performed the co-ordination of the study and statistical analysis and helped draft the manuscript. IB wrote the final draft of the manuscript. All authors read and approved the final version of the manuscript.

## Pre-publication history

The pre-publication history for this paper can be accessed here:

http://www.biomedcentral.com/1471-230X/12/36/prepub
